# MAOI Antidepressants: Could They Be a Next-Generation ICB Therapy? 

**DOI:** 10.3389/fimmu.2022.853624

**Published:** 2022-03-14

**Authors:** James Brown, Bo Li, Lili Yang

**Affiliations:** ^1^ Department of Microbiology, Immunology & Molecular Genetics, University of California, Los Angeles, Los Angeles, CA, United States; ^2^ Eli and Edythe Broad Center of Regenerative Medicine and Stem Cell Research, University of California, Los Angeles, Los Angeles, CA, United States; ^3^ Jonsson Comprehensive Cancer Center, The David Geffen School of Medicine, University of California, Los Angeles, Los Angeles, CA, United States; ^4^ Molecular Biology Institute, University of California, Los Angeles, Los Angeles, CA, United States

**Keywords:** monoamine oxidase inhibitor, immune checkpoint blockade, T cell, tumor-associated macrophage, immunotherapy, nanoformulation

## Introduction

The serotonin signaling pathway has been well understood in the neurons for decades (1, 2). During regular neuronal processes, serotonin released by the presynaptic neuron is used to communicate feelings of happiness and other behavioral changes with the postsynaptic neuron through binding to the 5-hydroxytryptamine receptor (5-HTR) (3, 4). After signaling, the presynaptic neuron reuptakes free serotonin to avoid overstimulation of 5-HTR, which is then broken down by the enzyme monoamine oxidase-A (MAO-A). The investigation of this critical neurotransmitter pathway has resulted in the development of many drugs capable of utilizing the diverse signaling regulators associated with serotonin to mitigate the effects of clinical depression and other neurological disorders. One of the first classes of small-molecule inhibitory drugs approved for the treatment of depression are MAO inhibitors (MAOIs), which prevent MAO-A from cleaving serotonin upon reuptake into the cells (3, 5). However, the MAO-A-serotonin signaling pathway outside of neurons is largely unknown. Recent discoveries have opened the door for understanding the function of MAO-A as an immunomodulatory molecule as well as examining its potential as a candidate for immune checkpoint blockade (ICB) therapy.

ICB therapy is the subject of growing interest as oncology is becoming an increasingly immunological field (6, 7). Anti-PD-1/PD-L1 therapy, which blocks PD-1-PD-L1 binding, circumventing T-cell exhaustion brought about by the immunosuppressive tumor microenvironment (TME), has been approved by the FDA as a treatment for solid tumors (5, 8). Another approved ICB therapy is anti-cytotoxic T lymphocyte-associated antigen 4 (anti-CTLA-4), which blocks the CTLA4 receptor from binding the apoptotic signaling ligand B7-1 (9). However, while these therapies have demonstrated significant or complete remission, factors such as multiple immune checkpoint pathways and their different roles in regulating specific cancer types can limit the efficacy of current ICB therapies (10). Therefore, it is of continuing interest to researchers that novel ICB candidates be developed in order to augment the effects of clinically-proven ICB through combination therapy that can improve immune cell infiltration and block suppressive pathways of tumor infiltrating immune cells (TIIs), increasing the tumor-type range and specificity of the field (10, 11). A number of ICB therapies are currently undergoing clinical trials, such as anti-CD47, which improves classically activated macrophage activity, as well as anti-Tim3 and anti-LAG3, which target common T cell negative immune checkpoints (12, 13). With the discovery of the serotonin pathway as a potential immune regulator, the promise of repurposing drugs that already have FDA approval as treatments for clinical depression offers the potential for a novel therapy that may increase the efficacy of current ICB treatments and broaden the reach of the field. This, in turn, makes the investigation of MAOIs as candidates of combination ICB therapy an incredibly interesting and exciting field of research with regards to cancer treatment.

## MAO-A as an Immune Regulator

**Figure 1:**
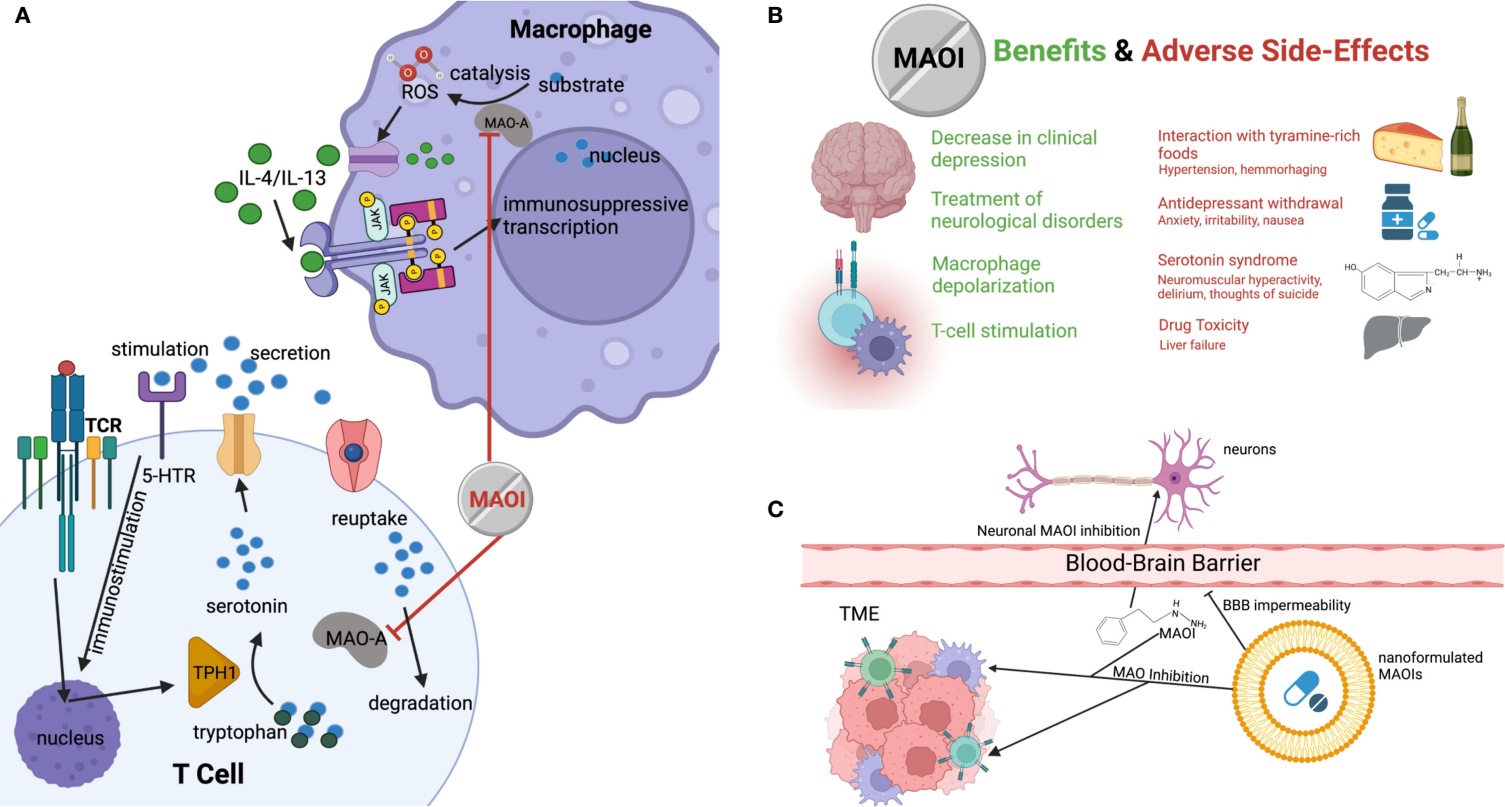
The potential of MAOI as a next-generation ICB therapy. **(A)** Diagram demonstrating how MAOIs inhibit MAO-A activity in immune cells, increasing free, immunostimulatory serotonin for intratumoral CD8 T cell stimulation and decreasing immunosuppression-inducing ROS produced by MAO-A oxidation of monoamine substrates in TAMs. **(B)** Current and potential clinical therapeutic benefits of MAOIs and the known associated side effects and symptoms. **(C)** Demonstration of nanoformulation of MAOIs preventing administered drugs from crossing the blood-brain barrier while maintaining ability to deliver drugs to target cells in tumor, avoiding the systemic application of the drug and mitigating side effects.

MAO-A was recently discovered to be upregulated in TIIs, especially in exhausted intratumoral CD8 T cells. This led to the hypothesis that MAO-A may be a negative regulator of anti-tumor T cell immunity through some unknown mechanism, so further studies examining the functions of knockout MAO-A T cells in tumor-challenged mice were conducted to determine whether there was any impact on oncolytic activity. Wang et al. reported that MAO-A-KO mice exhibited significantly suppressed tumor growth in two syngeneic mouse tumor models (5). This improved tumor-suppression response was accompanied by an increase in cytokine and cytotoxic molecule release in MAO-A-KO mice, indicating that the knockout of MAO-A results in increased cytotoxic lymphocyte (CTL) activity. To further dissect the mechanisms underlying the antitumor effects of MAO-A-KO T cells, researchers demonstrated that MAO-A-KO CD8 T cells produce higher levels of serotonin, which acts as an autocrine immune regulator to activate the downstream immunostimulatory mitogen-activated protein kinase (MAPK) pathway through 5-HTRs (5). This signaling pathway in turn “cross-talks” with the T cell receptor (TCR) signaling pathway, resulting in the enhanced effector function observed in MAO-A-KO T cells **(** **Figure 1A** **)** (5).

In addition to MAO-A’s role as a T cell regulator, another recent study revealed that MAO-A activity in tumor-associated macrophages (TAMs) was positively correlated with immune suppression (14). A knockout study comparing tumor-laden MAO-A-deficient mice and WT mice revealed that MAO-A-KO TAMs expressed lower levels of immunosuppressive markers and higher levels of immunostimulatory molecules (14). This led to the elucidation of a novel immunosuppressive mechanism of action associated with MAO-A in TAMs in addition to its role in T cells (5). The oxidation of monoamines in neurotransmitters such as serotonin by MAO-A results in the intracellular accumulation of reactive oxygen species (ROS) (14, 15). These ROS cause oxidative stress that polarizes the TME through the stimulation of the immunosuppressive JAK-Stat6 pathway and the production of immunosuppressive cytokines (**Figure 1A**) (16, 17). This demonstrated the multifaceted effects that MAO-A has on immune function in the TME.

## Potential Of MAOIs As ICB Therapy

MAO-A regulation of T cell activity was further investigated through the administration of MAOIs as a means of artificially inhibiting MAO-A activity and the breakdown of serotonin. In a mouse B16-OVA melanoma model, Wang et al. demonstrated dramatic tumor-suppressive effects of the MAOI antidepressants phenelzine, clorgyline, and moclobemide, which represented three classes of MAOIs, varying in the reversibility of the drug’s effects (reversible vs irreversible) and the selectivity for the serotonin-degrading MAO-A isoform over MAO-B (selective vs nonselective) (5). There was no observed significant difference in efficacy between the nonselective, reversible phenelzine; the selective, reversible moclobemide; and the nonselective, irreversible clorgyline, indicating that MAO-A specificity and drug irreversibility do not provide a therapeutic advantage with respect to antitumor response. Because phenelzine is commercially available in the United States, it was selected as a representative to determine whether MAOIs are a feasible candidate for ICB therapy. Phenelzine exhibited tumor-suppressive effects in mice of multiple mouse syngeneic tumor models (e.g. B16-OVA, MC38) as well as a human xenograft model (e.g. A375) (5). CTL antitumor activity was continuously demonstrated to be improved by the administration of phenelzine. In addition, phenelzine has been shown to exhibit tumor-suppressive effects in a stage II clinical trial by Gross et al. on patients with prostate cancer (18). Therefore, these findings suggest the translational potential of MAOIs as ICB therapy.

The efficacy of phenelzine as an ICB therapy was also demonstrated to inhibit immunosuppression through TAM polarization (14). This provides an interesting mechanism by which MAO-A inhibits the immune system’s natural antitumor abilities through the downregulation of ROS production, further demonstrating the immunological reasoning behind the efficacy of MAOIs as ICB candidates (**Figure 1A**). Strikingly, two reports demonstrated MAOI efficacy as both a monotherapy and combination therapy with anti-PD-1 in the typically responsive colon cancer MC38, as well as in B16 melanoma cells, which have historically been less responsive to immunotherapy (5). Phenelzine showed nearly identical efficacy to anti-PD-1, proving its utility as a monotherapy. What was even more promising, however, was the high significance in therapeutic improvement in the melanoma combination therapy model. This suggests that MAOIs may be able to drastically improve the reach of classical ICB therapies and opens the door for further testing to determine whether MAOIs have an effect in historically unresponsive cancer types such as breast, lung, prostate, and ovarian cancers.

## Possible Issues with MAOIs for ICB Therapy

While MAOIs were the primary class of antidepressants for the majority of the second half of the 20th century, they have fallen largely out of fashion due to the adverse side effects associated with their administration and the rise of alternative drugs (**Figure 1B**) (19). MAOIs have been known to cause severe dizziness due to hypertension, especially in elderly patients (20, 21). This presents an issue when considering MAOIs as cancer therapies for older patients, who are an important demographic for cancer treatments due to the high incidence of disease (22). These hypertensive side effects can also be made worse by an increase of dietary tyramine, which is typically metabolized by MAOs and can result in fatal brain hemorrhaging (23). Consequently, patients are often placed on restrictive diets excluding tyramine-rich foods such as cheese (hence the name “cheese effects”), making them a less appealing candidate for both neurological disorders and as ICB therapies when compared to other available options (14, 23). MAOIs have also been linked to significant liver toxicity when applied in high doses, making their long-term application less feasible when dealing with chronic illnesses (3). Additionally, MAOIs have been linked to serotonin syndrome when administered with other serotonergic drugs, which results from the accumulation of serotonin and can cause life threatening behavioral and physical maladies such as delirium, neuromuscular hyperactivity, and even thoughts of suicide (3, 20).

Despite these potential negatives, the utilization of MAOIs still shows significant promise, and the process should be optimized to increase drug efficacy and patient wellbeing. One potential source of side-effect remediation is through the development of nanoformulated MAOIs that can be targeted for delivery to the tumor. This would prevent many of the systemic side-effects of the drug and restrict its effects only to TIIs demonstrated to experience immunosuppression as a result of MAO-A activity (**Figure 1C**) (24). Liposomal drug delivery has been shown to limit systemic toxicity and improve drug delivery efficacy by inhibiting the diffusion of the nanoformulated particle across the blood-brain barrier (25). Currently, crosslinked multilamellar liposomes are the subject of ongoing trials for cancer drug delivery, and, by nanoformulating such liposomes for MAOI delivery, researchers may be able to increase drug payload size and decrease detrimental side effects, improving efficacy and avoiding life-threatening risks associated with these astounding, multifaceted drugs (26).

## Discussion

The discovery of MAO-A as an immune checkpoint offers an exciting new pathway that can be explored for the development of novel therapies repurposing drugs developed for the regulation of neuronal serotonin signaling (5, 14). Research has demonstrated that MAOIs have the ability not only to prevent the degradation of autocrine, immunostimulatory serotonin by MAO-A in T cells, but also to decrease immunosuppressive TAM polarization by inhibiting the production of ROS that results from MAO-A activity. Further studies are needed to investigate the clinical correlations between MAOI treatment and clinical outcomes in patients with cancer. Moreover, the identification and description of MAO-A as an immune checkpoint in CD8 T cells and TAMs suggests promising possibilities for investigation into its role as a checkpoint in other immune cell types.

While the potential associated with the use of a multifaceted ICB therapy such as MAOIs is impressive, it is important to consider the adverse side-effects that can greatly threaten the comfort and safety of the patient. However, there are several avenues of research that may help mitigate these side effects while successfully downregulating immunosuppressive pathways. As discussed, the use of liposomal drug delivery systems has shown significant promise in increasing the specificity of small-molecule drug delivery, and the development of a liposomal MAOI could provide the necessary specificity to avoid the adverse side effects currently associated with MAOI treatment (14, 18).

Additionally, the serotonergic pathway is complex, and, while the direct administration of serotonin would likely have limited lasting effects, other important protein targets in the serotonin production, reception, transport, and degradation pathway could potentially lead to more effective or less adverse ICB (5, 27). Because this pathway is well understood in the brain, there are already many types of drugs developed for the improvement of serotonin reception, several of which could potentially be repurposed for ICB therapy if demonstrated to have antitumor effects in future studies.

Because of the nature of cancer, novel drugs that can target the multifaceted pathways by which the disease avoids the immune system are always needed (6, 28). MAOIs in effect increase the amount of antitumor T cell stimulation by decreasing the expression of immunosuppressive markers *via* autologous serotonin-MAPK signaling, which is nonredundant to other immune checkpoint regulatory pathways, suggesting that they may have widespread applications as a combination therapy that increases the efficacy of classical ICBs. MAOIs’ inhibition of alternative macrophage polarization also plays a major role in increasing the host antitumor response (14). It remains to be seen whether combination with new ICB therapies which act directly on macrophage activity to promote immune infiltration, such as anti-CD47, increases patient response. In addition to their efficacy as an immune checkpoint blockade, MAOIs’ status as antidepressants could also be beneficial, as the dual role of this drug could help alleviate the depression experienced by many cancer patients while also assisting their immune system in combating their malignancy (14). While there is still much work to be done surrounding the full potential of serotonin regulation as an ICB therapy, the demonstration of MAOIs as an effective antitumor drug is a promising and inspiring development in the effort against cancer.

## Author Contributions

This manuscript was written by JB, BL, and LY. All authors contributed to the article and approved the submitted version.

## Funding

This work was supported by a Magnolia Council Senior Investigator Grant Award from the Tower Cancer Research Foundation (to LY).

## Conflict of Interest

L.Y. in an inventor on patents related to this topic filed by UCLA.

The remaining authors declare that the research was conducted in the absence of any commercial or financial relationships that could be construed as a potential conflict of interest.

## Publisher’s Note

All claims expressed in this article are solely those of the authors and do not necessarily represent those of their affiliated organizations, or those of the publisher, the editors and the reviewers. Any product that may be evaluated in this article, or claim that may be made by its manufacturer, is not guaranteed or endorsed by the publisher.

## References

[1] Pintar JE Breakefield XO. Monoamine Oxidase (MAO) Activity as a Determinant in Human Neurophysiology. Behavior Genetics (1982) 12:53–68. doi: 10.1007/BF01065740 6284115

[2] Deneris E Gaspar P. Serotonin Neuron Development: Shaping Molecular and Structural Identities. Wiley Interdiscip Rev: Dev Biol (2018) 7:1–26. doi: 10.1002/wdev.301 PMC5746461 29072810

[3] Bortolato M Chen K Shih JC. Monoamine Oxidase Inactivation: From Pathophysiology to Therapeutics. Adv Drug Deliv Rev (2008) 60:1527–33. doi: 10.1016/j.addr.2008.06.002 PMC2630537 18652859

[4] Jacobs BL Azmitia EC. Structure and Function of the Brain Serotonin System. Physiological Rev (1992) 72:165–230. doi: 10.1152/physrev.1992.72.1.165 1731370

[5] Wang X Li B Kim YJ Wang Y-C Li Z Yu J. Targeting Monoamine Oxidase a for T Cell-Based Cancer Immunother (2021). Available at: http://immunology.sciencemag.org/. 10.1126/sciimmunol.abh2383 33990379

[6] Vermaelen K Waeytens A Kholmanskikh O van den Bulcke M van Valckenborgh E. Perspectives on the Integration of Immuno-Oncology Biomarkers and Drugs in a Health Care Setting. Semin Cancer Biol (2018) 52:166–77. doi: 10.1016/j.semcancer.2017.11.011 29170067

[7] Chen DS Mellman I. Oncology Meets Immunology: The Cancer-Immunity Cycle. Immunity (2013) 39:1–10. doi: 10.1016/j.immuni.2013.07.012 23890059

[8] Page DB Postow MA Callahan MK Allison JP Wolchok JD. Immune Modulation in Cancer With Antibodies. Annu Rev Med (2014) 65:185–202. doi: 10.1146/annurev-med-092012-112807 24188664

[9] Wang H Kaur G Sankin AI Chen F Guan F Zang X. Immune Checkpoint Blockade and CAR-T Cell Therapy in Hematologic Malignancies. J Hematol Oncol (2019) 12:1–20. doi: 10.1186/s13045-019-0746-1 31186046 PMC6558778

[10] Yeon Yeon S Jung SH Jo YS Choi EJ Kim MS Chung YJ. Immune Checkpoint Blockade Resistance-Related B2M Hotspot Mutations in Microsatellite-Unstable Colorectal Carcinoma. Pathol Res Pract (2019) 215:209–14. doi: 10.1016/j.prp.2018.11.014 30503610

[11] Ngiow SF Young A Jacquelot N Yamazaki T Enot D Zitvogel L. A Threshold Level of Intratumor CD8+ T-Cell PD1 Expression Dictates Therapeutic Response to Anti-PD1. Cancer Res (2015) 75:3800–11. doi: 10.1158/0008-5472.CAN-15-1082 26208901

[12] Zhang X Chen W Fan J Wang S Xian Z Luan J. Disrupting CD47-Sirpα Axis Alone or Combined With Autophagy Depletion for the Therapy of Glioblastoma. Carcinogenesis (2018) 39:689–99. doi: 10.1093/carcin/bgy041 29538621

[13] Patel SA Minn AJ. Combination Cancer Therapy With Immune Checkpoint Blockade: Mechanisms and Strategies. Immunity (2018) 48:417–33. doi: 10.1016/j.immuni.2018.03.007 PMC6948191 29562193

[14] Wang YC Wang X Yu J Ma F Li Z Zhou Y. Targeting Monoamine Oxidase a-Regulated Tumor-Associated Macrophage Polarization for Cancer Immunotherapy. Nat Commun (2021) 12:1–17. doi: 10.1038/s41467-021-23164-2 34112755 PMC8192781

[15] Ding F Liu B Zou W Tian D Li Q Dai J. LPS Exposure in Early Life Protects Against Mucus Hypersecretion in Ovalbumin-Induced Asthma by Down-Regulation of the IL-13 and JAK-STAT6 Pathways. Cell Physiol Biochem (2018) 46:1263–74. doi: 10.1159/000489109 29680833

[16] Nappo G Handle F Santer FR McNeill R v. Seed RI Collins AT. The Immunosuppressive Cytokine Interleukin-4 Increases the Clonogenic Potential of Prostate Stem-Like Cells by Activation of STAT6 Signalling. Oncogenesis (2017) 6:1–12. doi: 10.1038/oncsis.2017.23 PMC5523058 28553931

[17] Charras A Arvaniti P le Dantec C Dalekos GN Zachou K Bordron A. JAK Inhibitors and Oxidative Stress Control. Front Immunol (2019) 10:2814. doi: 10.3389/fimmu.2019.02814 31867003 PMC6908489

[18] Gross ME Agus DB Dorff TB Pinski JK Quinn DI Castellanos O. Phase 2 Trial of Monoamine Oxidase Inhibitor Phenelzine in Biochemical Recurrent Prostate Cancer. Prostate Cancer Prostatic Dis (2021) 24:61–8. doi: 10.1038/s41391-020-0211-9 PMC7483294 32123315

[19] Tran BX Ha GH Vu GT Nguyen LH Latkin CA Nathan K. Indices of Change, Expectations, and Popularity of Biological Treatments for Major Depressive Disorder Between 1988 and 2017: A Scientometric Analysis. Int J Environ Res Public Health (2019) 16:1–15. doi: 10.3390/ijerph16132255 PMC6651662 31247926

[20] Sabri MA Saber-Ayad MM. Mao Inhibitors. Treasure Island, FL: StatPearls Publishing (2021). 32491327

[21] Volz H-P Gleiter CH. Monoamine Oxidase Inhibitors a Perspective on Their Use in the Elderly. Drugs & Aging (1998) 13:341–55. doi: 10.2165/00002512-199813050-00002 9829163

[22] Ellison EC Pawlik TM Way DP Satiani B Williams TE. The Impact of the Aging Population and Incidence of Cancer on Future Projections of General Surgical Workforce Needs. Surg (United States) (2018) 163:553–9. doi: 10.1016/j.surg.2017.09.035 29179915

[23] Carradori S Secci D Petzer JP. MAO Inhibitors and Their Wider Applications: A Patent Review. Expert Opin Ther Patents (2018) 28:211–26. doi: 10.1080/13543776.2018.1427735 29324067

[24] Mancini S Nardo L Gregori M Ribeiro I Mantegazza F Delerue-Matos C. Functionalized Liposomes and Phytosomes Loading Annona Muricata L. Aqueous Extract: Potential Nanoshuttles for Brain-Delivery of Phenolic Compounds. Phytomedicine (2018) 42:233–44. doi: 10.1016/j.phymed.2018.03.053 29655691

[25] Rip J Chen L Hartman R van den Heuvel A Reijerkerk A van Kregten J. Glutathione Pegylated Liposomes: Pharmacokinetics and Delivery of Cargo Across the Blood-Brain Barrier in Rats. J Drug Targeting (2014) 22:460–7. doi: 10.3109/1061186X.2014.888070 PMC4651142 24524555

[26] Joo K Xiao L Liu S Liu Y Lee CL Conti PS. Crosslinked Multilamellar Liposomes for Controlled Delivery of Anticancer Drugs. Biomaterials (2013) 34:3098–109. doi: 10.1016/j.biomaterials.2013.01.039 PMC3995748 23375392

[27] Taciak PP Lysenko N Mazurek AP. Drugs Which Influence Serotonin Transporter and Serotonergic Receptors: Pharmacological and Clinical Properties in the Treatment of Depression. Pharmacol Rep (2018) 70:37–46. doi: 10.1016/j.pharep.2017.07.011 29309998

[28] Chowdhury PS Chamoto K Honjo T. Combination Therapy Strategies for Improving PD-1 Blockade Efficacy: A New Era in Cancer Immunotherapy. J Internal Med (2018) 283:110–20. doi: 10.1111/joim.12708 29071761

